# Liver Fibrosis Scores and Hospitalization, Mechanical Ventilation, Severity, and Death in Patients with COVID-19: A Systematic Review and Dose-Response Meta-Analysis

**DOI:** 10.1155/2022/7235860

**Published:** 2022-03-29

**Authors:** Menglu Liu, Kaibo Mei, Ziqi Tan, Shan Huang, Fuwei Liu, Chao Deng, Jianyong Ma, Peng Yu, Xiao Liu

**Affiliations:** ^1^Department of Cardiology, The Seventh People's Hospital of Zhengzhou, Zhengzhou, Henan, China; ^2^Department of Anesthesiology, The People's Hospital of Shanggrao, Shanggrao, Jiangxi, China; ^3^Department of Endocrine, The Second Affiliated Hospital of Nanchang University, Nanchang, Jiangxi, China; ^4^Department of Psychiatry, The Third People's Hospital of Gan Zhou, Ganzhou, Jiangxi, China; ^5^Department of Cardiology, The Affiliated Ganzhou Hospital of Nanchang University, Ganzhou, Jiangxi, China; ^6^Department of Cardiology, The Affiliated Hospital of Jiangxi University of Chinese Medicine, Nanchang, Jiangxi, China; ^7^Department of Pharmacology and Systems Physiology, University of Cincinnati College of Medicine, Cincinnati, OH, USA; ^8^Institute for the Study of Endocrinology and Metabolism in Jiangxi, Nanchang, China

## Abstract

**Methods:**

We identified relevant cohort studies that assessed the relationship between liver fibrosis scores (e.g., FIB-4, NAFLD fibrosis score (NFS), and aspartate aminotransferase to platelet ratio index (APRI)) and associated prognosis outcomes by searching the PubMed, EMBASE, and medRxiv databases. The potential dose-response effect was performed using a stage robust error meta-regression.

**Results:**

Sixteen studies with 8,736 hospitalized patients with COVID-19 were included. One-point score in FIB-4 increase was significantly associated with increased mechanical ventilation (RR: 2.23, 95% CI: 1.37–3.65, *P*=0.001), severe COVID-19 (RR: 1.82, 95% CI: 1.53–2.16, *P* < 0.001), and death (RR: 1.47, 95% CI: 1.31–1.65, *P* < 0.001), rather than hospitalization (RR: 1.35, 95% CI: 0.72–2.56, *P*=0.35). Furthermore, there is a significant positive linear relationship between FIB-4 and severe COVID-19 (*P*_nonlinearity_=0.12) and mortality (*P*_nonlinearity_=0.18). Regarding other liver scores, one unit elevation in APRI increased the risk of death by 178% (RR: 2.78, 95% CI: 1.10–6.99, *P*=0.03). Higher NFS (≥−1.5) and Forns index were associated with increased risk of severe COVID-19 and COVID-19-associated death.

**Conclusion:**

Our dose-response meta-analysis suggests high liver fibrosis scores are associated with worse prognosis in patients with COVID-19. For patients with COVID-19 at admission, especially for those with coexisting chronic liver diseases, assessment of liver fibrosis scores might be useful for identifying high risk of developing severe COVID-19 cases and worse outcomes.

## 1. Introduction

Chronic liver diseases occur very commonly worldwide and have become one of the major global health burdens [1]. Hepatic fibrosis is the early histological change before the development of cirrhosis which is the end sequela in many liver diseases (e.g., hepatitis B or hepatitis C virus infection, chronic alcoholism, and nonalcoholic fatty liver disease (NAFLD)) [2]. Noninvasive liver fibrosis scores have been developed to screen the extent of liver fibrosis (e.g., fibrosis-4 (FIB-4), NAFLD fibrosis score (NFS), and aspartate aminotransferase to platelet ratio index (APRI)) in chronic liver diseases and validated to use as prognostic indicators [3,4], for NAFLD [5,6], liver cancer [7], and patients infected with chronic hepatitis virus [8]. Moreover, they were also identified as diagnostic indicators in other population, such as the general population or patients with established cardiovascular diseases [6,9].

Coronavirus disease 2019 (COVID-19), which is caused by SARS-CoV-2, resulted in over 5 million deaths worldwide. Accumulating evidence suggests that COVID-19 is more than a respiratory disease. Broad spectra of extrapulmonary manifestations, including heart, liver, and microvascular injuries, were also widely observed in patients with COVID-19. These extrapulmonary manifestations served as the strongest predictors for severity and mortality due to COVID-19 [10,11]. With the ongoing COVID-19 pandemic, preexisting chronic liver diseases are found to be one of the highest prevalent comorbidities [12]. Ji et al. reported that the NAFLD has been reported in up to 38% of patients with COVID-19, and it has been associated with a worse prognosis [13,14]. Moreover, the liver fibrosis score that assesses the advanced fibrosis (e.g., FIB-4 and NFS) was also correlated with increased risk for mechanical ventilation (MV), intensive care, and mortality [15,16]; however, with inconsistent results [17–19]. Furthermore, we noted that the liver fibrosis scores and clinical outcomes in patients with COVID-19 were not comprehensively assessed. Given these circumstances, this systematic review and meta-analysis aimed to evaluate the relationship between liver fibrosis scores and adverse outcomes in patients with COVID-19, as well as potential dose-response association.

## 2. Methods

This study is a PRISMA-compliant (2021) systematic review and meta-analysis [20]. In addition, the protocol was prospectively registered with the international prospective register of systematic reviews (PROSPERO), and the registration number is CRD42021265872 ( see Supplementary Table S1).

### 2.1. Search Strategy

Four databases such as PubMed, Embase, medRxiv, and Cochrane Library were initially searched, up to June 5^th^ 2021. The search terms on liver fibrosis scores (such as FIB-4, NFS, and APRI) and clinical outcomes (hospitalization, MV, intensive care unit (ICU) admission, severe COVID-19, and mortality) in patients with COVID-19 were used with no language restriction. The full search strategy was described in detail in Supplementary Table S2. In addition, the reference lists of the relevant articles or reviews were further explored.

### 2.2. Selection Criteria and Study Selection

We included articles that met the following criteria: (1) studies reporting the associated clinical outcomes (hospitalization, mechanical ventilation (MV), severe COVID-19, and death) with noninvasive liver fibrosis scores in adult patients with COVID-19; (2) elucidations reporting the adjusted estimate (odds ratio (OR), risk ratio (RR), or hazard ratio (HR)) and corresponding 95% CI of the relevant outcomes; (3) cohort studies. Case reports, case-serial reports, comments, and reviews were excluded from the analysis. Furthermore, case-control studies and articles reporting unadjusted results were excluded to reduce bias. Two authors (XL and PY) independently conducted the above process, and inconsistencies were rectified by discussing with the third author.

### 2.3. Data Collection and Quality Assessment

Data were extracted based on the prespecified inclusion criteria. The following information was abstracted: study characteristics (first author's name, publication year, country in which the study was conducted, and study design), patient characteristics (sample size, age, and sex), exposures (number of fibrosis cases), and outcomes (number of events, adjusted ORs/RRs/HRs and the corresponding 95% CI, and adjustments).

The Newcastle–Ottawa quality scale (NOS) was applied to assess the quality of nonrandomized studies. Studies with a NOS of ≥6 stars were considered as moderate to high-quality articles [21].

### 2.4. Statistical Analysis

We used the random effect model to make our results more reliable, considering the potential heterogeneity. The study-specific RRs and 95% CIs for one-point increment in liver fibrosis scores were calculated using the Greenland and Longnecker method [22]. The nonlinear dose-response relationship was fitted following the method described by Xu and Doi [23]. It requires at least two levels of quantitative exposure categories and the corresponding RRs and variance estimates [23]. If the liver fibrosis score was not directly reported or reported in ranges, we estimated the midpoint of each category by averaging the lower and upper boundaries of that category [24,25]. If the highest or lowest class was open-ended, we assumed that the open-ended interval length was the same as the adjacent interval [26]. In this study, the OR and HR were equally treated as RR according to our previous articles [22]. ICU admission was also defined as severe COVID-19 as we previously described [27]. We evaluated the degree of heterogeneity among the studies included in the analysis using the *I*^*2*^ test (25%, 50%, and 75% represent low, moderate, and high heterogeneity, respectively) [27,28]. Sensitivity analyses were performed by omitting each study in turn. Stata software (version 16.0) and RevMan software (version 5.3, Cochrane Collaboration, Nordic Cochrane Center Copenhagen, Denmark) were used for statistical analysis. All statistical tests were double-sided, and *P* < 0.05 considered statistically significant.

## 3. Results

### 3.1. Study Selection

**Figure 1:**
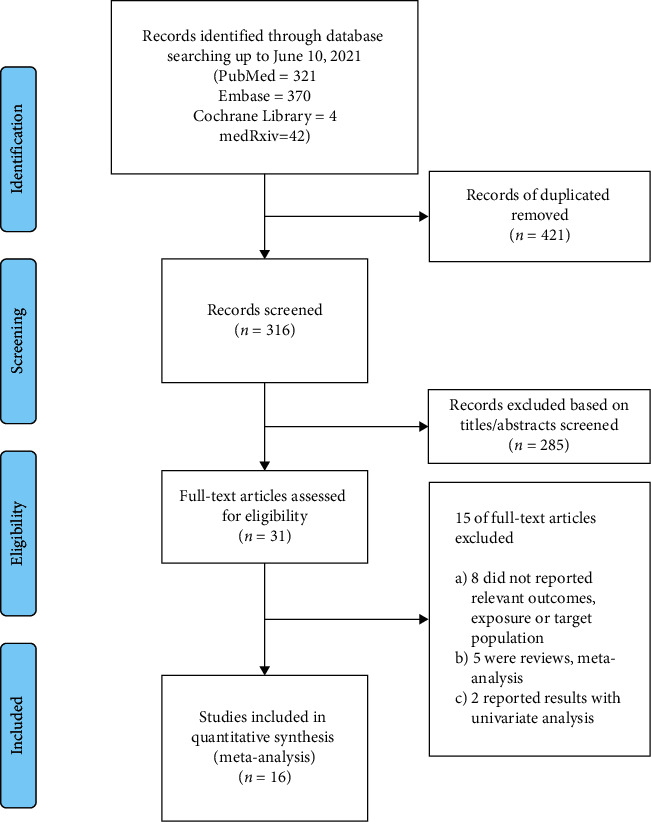
Flow chart of this meta-analysis.

As shown in Figure 1, 1737 studies were initially retrieved by searching the PubMed, Cochrane Library, medRxiv, and Embase databases. We excluded 421 duplicated records and 285 articles, which were not relevant to the study objective after reviewing the title and abstract. Sixteen articles [15,16,18,19,29–40] were finally included after excluding 15 reports for the following reasons: (1) reports that did not report the relevant clinical outcomes or target population (*N* = 8), (2) elucidations that were case reports or consisted only of comments (*N* = 5), and (3) studies that reported results with an unadjusted estimate effect (*N* = 2). The detailed exclusion criteria for each study are described in Supplementary Table S3.

### 3.2. Study Characteristics and Study Quality

The basic characteristics of the studies included are described in Table 1. Overall, sixteen cohorts (fifteen retrospective [15–19,29–34,36–40] and one prospective [35]) involving 8,736 hospitalized patients with COVID-19 were included. All COVID-19 cases were diagnosed by real-time PCR. The mean age ranged from 47 to 72 years, and five reports were from the US. Five studies were from Europe, and six publications were from Asia. Ten [15,16,18,29,31–34,36,38] articles reported FIB-4, three [30,35,40] reported aspartate aminotransferase (AST)/alanine aminotransferase (ALT) ratio, one [39] reported NFS, one reported FIB-4 and NFS [37], and one [19] assessed FIB-4 and Forns index score. All studies were found to be acceptable (*N* ≥ 6) elucidations assessed by the NOS (see Supplementary Table S4).

### 3.3. Dose-Response Relationship between FIB-4 and Clinical Outcomes in COVID-19

**Figure 2:**
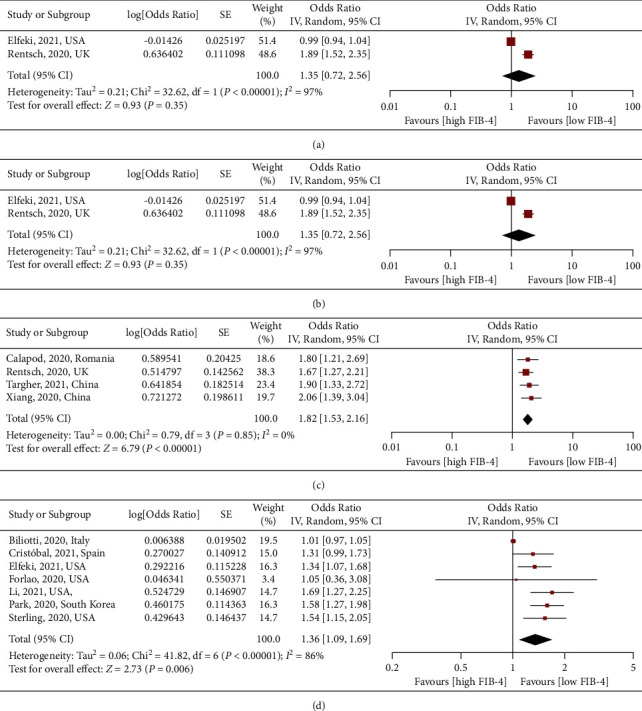
Association between FIB-4 and clinical outcomes in patients with COVID-19. FIB-4 was analyzed for continuous analysis (per one-point increase). (a) Hospitalization. (b) MV. (c) Severe COVID-19. (d) Death. Abbreviation: MV, mechanical ventilation.

**Figure 3:**
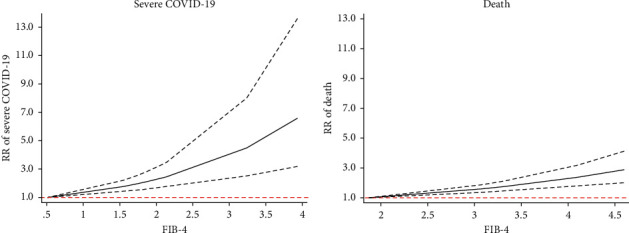
Nonlinear exposure-effect analysis between liver fibrosis scores and clinical outcomes in patients with COVID-19. (a) Hospitalization. (b) MV. (c) Severe COVID-19. (d) Death. The solid and dashed lines represent the estimated odd risk and the 95% confidence interval, respectively. Abbreviation: MV, mechanical ventilation.

Thirteen [15–29, 31–34, 36–38] studies reported FIB-4 and associated clinical outcomes in patients with COVID-19. Two studies reported hospitalization, two elucidations reported MV, five studies reported severity, and six studies reported death (Table 1). As shown in Figure 2, one-point score increase in FIB-4 was significantly associated with the increased MV (RR: 2.23, 95% CI: 1.37–3.65, *P*=0.001, I2 = 0%), severe COVID-19 (RR: 1.82, 95% CI: 1.53–2.16, *P* < 0.001, I2 = 0%), and death (RR: 1.47, 95% CI: 1.31–1.65, *P* < 0.001, I2 = 0%), rather than hospitalization (RR: 1.35, 95% CI: 0.72–2.56, *P*=0.35, I2 = 0%). All the pooled results showed no evidence of heterogeneity. In addition, there was a linear association between FIB-4 and severe COVID-19 (*P*_nonlinearity_=0.12) and death (*P*_nonlinearity_=0.18) in patients with COVID-19 (Figure 3).

### 3.4. Association between Other Liver Fibrosis Scores and Clinical Outcomes in COVID-19

Three studies reported an association between the AST/ALT ratio and death. The results showed that one unit elevation in AST/ALT ratio increased the risk of death by 178% (RR: 2.78, 95% CI: 1.10–6.99, *P*=0.03, I2 = 76%). The heterogeneity was not significant when excluding the study by Sarin et al., and the results did not change (RR: 4.51, 95% CI: 1.59–12.77, *P*=0.005, I2 = 38%). Targher et al. [37] reported that higher NFS (≥−1.5) increased the risk of developing severe COVID-19 by ten-fold after adjustments. Romero-Cristobal et al. [19] showed that a one-point increment in the Forns index increased the risk of death by 41% through a multivariate analysis.

### 3.5. Publication Bias and Sensitive Analysis

Publication bias was not evaluated because of the limited number of studies according to the guideline (*N* < 10) [41]. The results were stable in the sensitive analysis by omitting one study at a time (Supplementary Figure S1).

## 4. Discussion

To our best of knowledge, this is the first comprehensive meta-analysis that assessed the live fibrosis scores and clinical outcomes in patients with COVID-19, as well as the potential dose-response relationship. Based on current evidence, we showed that all available liver fibrosis scores, including FIB-4, Forn, NFS, and AST/ALT ratio, were associated with a worse prognosis in patients with COVID-19. Moreover, there was a positive linear relationship between the FIB-4 and severe COVID-19 and death.

Several noninvasive methods were developed using serum biomarkers (e.g., FIB-4, NFS, and APRI) to assess liver fibrosis [42]. Previous studies have shown that liver fibrosis is associated with increased mortality due to cardiovascular risk and all-cause mortality in patients with liver diseases or the general population [9,43,44]. In the present study, we found a positive association between liver fibrosis scores and adverse outcomes. These results were consistent with the recent findings, which reported worse outcomes in COVID-19 patients with preexisting chronic liver diseases [45]. For example, FIB-4 was found to be an independent factor of mortality among hospitalized COVID-19 patients with imaging- or liver biopsy-proven NAFLD [46]. Sachdeva et al. found that the NFLAD was a strong predictor for mortality in patients infected with SARS-CoV-2 [13,45]. It should be noted that the prevalence of chronic liver diseases is low (3%) in previous pooled analysis [47], which might be vastly underestimated. The rate of liver fibrosis assessed by the liver fibrosis score is much larger than the prevalence of chronic liver diseases. For example, the cohort in the study by Sterling et al. had a high frequency of increased FIB-4 (52% had a FIB-4 level of >2.67 and 42% had a FIB-4 level of >3.25); however, there was low prevalence of known underlying liver disease (6%). In general, FIB-4 or NFS scores have shown higher negative predictive value but lower positive predictive value. That is to say, they have better accuracy in excluding rather than in identifying advanced fibrosis. The presence of advanced fibrosis might be underestimated in COVID-19 patients. Therefore, these liver fibrosis scores provide valuable information for patients with liver comorbidities with COVID-19 and can be an effective prognostic marker for predicting their prognosis.

Moreover, the current evidence shows that FIB-4 and NFS did not perform accurately in some population, such as younger patients (< 35 years) and lean and morbidly obese adults [48,49]. The average mean age and BMI of included studies ranged from 47 to 72 years and 24.1 to 30.8 kg/m^2^, respectively. Moreover, subgroup analyses stratified by mean age and mean BMI cannot be performed due to data restriction. The prognosis role of liver fibrosis in the COVID-19 population should be further studied.

It should be pointed out that these noninvasive assessments should be interpreted with caution due to more complexities during COVID-19 progression. Apart from the underestimated prevalence of NAFLD, we speculated that the elevation of these indicators was likely due to multiple factors but was linked to the COVID-19 disease pathogenesis and severity [10,11]. Muscular injuries and hepatocellular and portal system alterations due to SARS-CoV-2 infection and systemic inflammation play a role in these outcomes. The components of these scores, such as AST, ALT, and platelet levels, largely fluctuated with the natural history of COVID-19 [32]. Several studies showed that the AST and ALT were significantly increased due to the high incidence of liver injury in COVID-19 patients [50]. The FIB-4 level was correlated to SARS-CoV-2 plasma RNA level as well as monocyte-associated cytokine levels [32]. Therefore, we should figure out whether the prevalence of liver fibrosis in COVID-19 can be solely attributed to chronic liver diseases and whether the associated incidence of liver injury can be caused by COVID-19.

There might be several potential mechanisms in the pathogenesis of chronic liver disease. Inflammation has a vital role in the pathogenesis of liver fibrosis [51]. Chronic inflammation is firmly established, and advanced liver disease is characterized by low-grade systemic inflammation caused by activated immune cells [51]. These activated cells serve as a vital source of cytokines and chemokines (e.g., interleukin-6, interleukin-18, and interleukin-17). Furthermore, Li et al. showed a positive association between FIB-4 scores and interleukin-6 levels in patients with COVID-19 [32]. Some researchers proposed that this elevated interleukin-6, which is partly secreted by activated macrophages induced during liver fibrosis, might induce inflammatory response proteins in the hepatocytes (such as CRP (C-reactive protein), ferritin, complement, and clotting factors) [31]. Meanwhile, as it is known, an excessive inflammatory response is a relative phenomenon of severe COVID-19 cases. Therefore, it is reasonable to speculate that liver fibrosis may increase the risk of exacerbated inflammatory responses.

Overall, our results showed that the liver fibrosis scores were associated with the worst prognosis and might be a simple marker for predicting the severity and mortality in patients with COVID-19. All the components of these liver fibrosis scores (e.g., age, AST, and ALT) were accessible, and determining the levels of these markers was inexpensive. However, importantly, we did not assess the correlation between the presence of fibrosis and the most accurate assessment test, liver biopsy. Admittedly, liver biopsy is the current gold standard test for assessing liver fibrosis. However, it is unfeasible, probably unethical, and difficult to perform routinely. Furthermore, liver fibrosis scores were the results of multiple and complex factors involved in the natural progression of SARS-CoV-2 infection and should not be merely considered as an assessment for liver fibrosis.

### 4.1. Strength and Limitation

This is the first meta-analysis to comprehensively assess the liver fibrosis scores and associated clinical outcomes in patients with COVID-19 and elucidate the positive linear association between the FIB-4 and adverse outcomes. Our study inevitably has several limitations. Firstly, this is an analysis of observational research, which cannot prove causation. Secondly, the number of studies included was relatively limited, and prospective, longitudinal, larger studies were needed to validate the predictive ability of liver fibrosis scores. Thirdly, as the components of liver fibrosis scores varied during trajectories of COVID-19, the inconsistent timepoint of assessment included in evaluations, the studies inevitably increased the instability of predicting adverse outcomes in patients with the COVID-19. Fourthly, the specificity of FIB-4 for determining advanced fibrosis in patients ≥65 years decreases significantly and may overestimate the liver fibrosis level [49]. However, we cannot perform a subgroup stratified analysis by age. Further studies should focus on determining if there is an age difference.

## 5. Conclusion

Overall, our results suggested that liver fibrosis scores, such as FIB-4, NFS, AST/ALT ratio, and Forns index were significantly associated with the increased risk of MV, severe COVID-19, and mortality. For patients with COVID-19 at admission, especially for those with coexisting chronic liver diseases, assessment of liver fibrosis scores might be useful for identifying high risk of developing severe COVID-19 cases and worse outcomes.

## Figures and Tables

**Table 1:** Baseline characteristics of included studies for the association between liver scores and clinical outcomes in patients with COVID-19.

Author, year, country	Study design	Sample size	Population	Data source	Age, female	Liver score reported (outcomes)	Estimate effect	Adjustments
Xiang, 2020 [38], China	Retrospective cohort	267	COVID-19	Guangzhou No. 8 People's hospital	47, 54	FIB-4 (IMV)		Sex, hypertension, DM, heart diseases, liver diseases, kidney diseases, psychological disorders, time from admission to symptom onset date, D-dimer, and CRP
						<1.45	1	
						1.45–3.25	4.18 (0.39–45.23)	
						>3.25	10.16 (0.80–128.51)	
						FIB-4 (severe COVID-19)		
						<1.45	1	
						1.45–3.25	4.63 (1.47–14.58)	
						>3.25	11.92 (3.14–45.20)	
Cristóbal, 2021[19] Spain	Retrospective cohort	214	COVID-19 admitted in ICU	Hospital General Universitario Gregorio Marañón	59, 28	FIB-4 (death)		Charlson comorbidity index, the acute physiology and chronic health evaluation II, and serum ferritin
						Per 1 unit	1.31 (0.99–1.72)	
						Forns	1.41 (1.11–1.81)	
Elfeki, 2021[16], USA	Retrospective cohort	373	COVID-19 with metabolic syndrome	UnityPoint Clinic or Hospital in the state of Iowa	62, 48	FIB-4 (death)		Type 2 DM and CKD
						<1.30	1	
						1.30–2.67	1.52 (0.37–6.34)	
						>2.67	2.22 (1.20–4.12)	
						FIB-4 (hospitalization)		
						<1.30	1	
						1.30–2.67	1.67 (1.06–2.64)	
						>2.67	0.96 (0.84–1.10)	
Samaniego, 2021[31], Spain	Retrospective cohort	160	COVID-19	5 tertiary-level hospitals in the region of Madrid	55, 66	FIB-4 (severe COVID-19)		Hypertension, respiratory disease, and bilirubin, LDH acute C-reactive protein
						<1.30	1	
						≥ 2.67	3.41 (1.30–8.92)	
Li, 2021[32], USA,	Retrospective cohort	202	COVID-19	Two large academic centers in Boston, Massachusetts	58, 46	FIB-4 (death)		Sex, BMI, ethnicity, hypertension, diabetes, remdesivir use, and history of liver diseases, baseline troponin *T*, CRP, lymphocyte count, LDH, and D-dimer
						<2.67	1	
						≥2.67	6.29 (2.10–18.80)	
						Per 1 unit	1.63 (1.22–2.17)	
Calapod, 2020[15], Romania	Prospective cohort	138	COVID-19 with type II DM	Bucharest Emergency University	66, 42	FIB-4 (severe COVID-19)		Sex, BMI, dyspnea, ferritin, CRP, AST, and ALT
						<1.30	1	
						1.30–2.67	2.47 (1.01–7.63)	
						>2.67	4.89 (1.34–12.3)	
Forlano, 2020[18], USA	Retrospective cohort	193	COVID-19 with NAFLD	Imperial College Healthcare NHS Trust	66, 67	FIB-4 (death)		Male, presence of type 2 DM, hypertension, dyslipidemia
						<3.5	1	
						≥3.25	1.07 (0.15–3.5)	
Targher, 2021[37], China	Retrospective cohort	310	NAFLD	Four sites in Zhejiang province	48, 62	FIB-4 (severe COVID-19)		Sex, obesity, diabetes, and presence/absence of MAFLD
						No MAFLD	1	
						<1.3	0.82 (0.30–2.24)	
						≥1.3	2.95 (1.37–6.34)	
						FIB-4 (severe COVID-19)		
						Per 1 unit	1.90 (1.33–1.72)	
						NFS (severe COVID-19)		
						Per 1 unit	2.57 (1.73–3.82)	
Park, 2020[33], South Korea	Retrospective cohort	1005	COVID-19	Five tertiary hospitals of Daegu	72, 54	FIB-4 (death)		DM, COPD, lymphocyte count, e-GFR, SIRS on admission
						<4.95	1	
						≥4.95	2.78 (1.69–4.58)	
Sterlin,2020[36], USA	Retrospective cohort	256	COVID-19	Virginia Commonwealth University Medical Center in Richmond	58, 45	FIB-4 (death)		DM, kidney, cardiovascular diseases, and respiratory diseases
						<2.67	1	
						≥2.67	1.68 (1.19–2.38)	
						FIB-4 (IMV)		
						<2.67	1	
						≥2.67	3.09 (1.38–6.93)	
Rentsch, 2020[34], UK	Retrospective cohort	3,789	COVID-19	VA National Corporate Data Warehouse on Members of the VA Birt	65, 10	FIB-4 (hospitalization)		Race, CKD, COPD, DM, hypertension, vascular disease, ACEI/ARB, NASIDs, SBP, oxygen saturation, albumin, e-GFR, hemoglobin, white blood cell count, lymphocyte count, VACS index score#
						<1.45	1	
						1.45–3.25	2.96 (1.69–5.17)	
						>3.25	8.73 (4.11–18.56)	
						FIB-4 (severe COVID-19)		
						<1.45	1	
						1.45–3.25	4.59 (1.72–12.22)	
						>3.25	8.40 (2.90–24.28)	
Yao, 2021[39], China	Retrospective cohort	342	RT-PCR	Hospitals of Jiangsu province		NFS (severe COVID-19) <−1.5 ≥−1.5	Ref. 11.05 (1.19,102.43)	Age, gender, BMI, hypertension, diabetes
Biliotti, 2020[29], Italy	Retrospective cohort	299	COVID-19	INMI Lazzaro Spallanzani	54	FIB-4 (ICU admission or death) <2.67 ≥2.67	Ref. 1.35 (1.04–1.75)	Presence of severe pneumonia, obesity, and C- reactive protein
Fu, 2020[40], China	Case-cohort	200	COVID-19	Second Affiliated Hospital of Anhui Medical University	50.7, NA	AST/ALT (death) per 1	3.22 (1.59, 6.56)	Total bilirubin, alanine aminotransferase, creatinine, urea nitrogen, uric acid, creatine kinase, myoglobin, lactate dehydrogenase, aspartate aminotransferase
Sarin, 2020[35], international	Retrospective cohort	228	COVID-19 with preexisting chronic liver disease	APASL-ACLF Research Consortium Registry Study	51,47	AST/ALT (death) per 1	1.4 (2.5–5.4)	Total bilirubin
Goel, 2020[30], USA	Retrospective cohort	551	COVID-19	St Luke's University Hospital	63, NA	AST/ALT (death) per 1	2.75 (1.63–4.65)	Age, hypertension, diabetes, heart failure, chronic kidney disease, malignancy, chronic pulmonary disease, and chronic liver disease, total bilirubin, and the inflammatory marker

COPD: chronic obstructive lung disease; CKD: chronic kidney diseases; NASIDs: nonsteroidal anti-inflammatory drugs; MAFLD, metabolic dysfunction-associated fatty liver disease; e-GFR, estimated glomerular filtration rate; ACEI/ARB: angiotensin converting enzyme inhibitor/ angiotensin receptor blocker; BMI, body mass index; DM: diabetes mellitus; SBP: systolic blood pressure; DBP: diastolic blood pressure; CRP: C-reactive protein; AST: aspartate aminotransferase; ALT: alanine aminotransferase. #The VACS Index score is a validated measure of physiologic injury combining age, aspartate and alanine transaminase, albumin, creatinine, hemoglobin, platelets, white blood cell count, hepatitis C status, and body mass index.

## Data Availability

The datasets used and analyzed during the current study are available from the corresponding author on reasonable request.

## Supplementary Materials

Supplementary Materials Figure S1: sensitive analysis between liver fibrosis scores and clinical outcomes in patients with COVID-19 by omitting one study at a time. A: severe COVID-19; B: death. Supplementary Table S1: PRISMA 2020. Table S2: search strategy. Table S3: studies excluded with reasons. Supplemental Table S4: Newcastle–Ottawa Scale (NOS) scores for included studies.
