# Supplementary material for: Liver Fibrosis Scores and Hospitalization, Mechanical Ventilation, Severity, and Death in Patients with COVID-19: A Systematic Review and Dose-Response Meta-Analysis
Source: Can J Gastroenterol Hepatol. 2022 Mar 29;2022:7235860. doi: 10.1155/2022/7235860 (PMC8966744; doi:10.1155/2022/7235860)
Supplement: Supplementary Materials — Figure S1: sensitive analysis between liver fibrosis scores and clinical outcomes in patients with COVID-19 by omitting one study at a time. A: severe COVID-19; B: death. Supplementary Table S1: PRISMA 2020. Table S2: search strategy. Table S3: studies excluded with reasons. Supplemental Table S4: Newcastle–Ottawa Scale (NOS) scores for included studies. [file 7235860.f1.docx]

**Association between liver fibrosis scores and clinical outcomes in patients with COVID-19:**

**A systematic review and meta-analysis**

**Supplemental Table S1: PRISMA 2020**

| **Section and Topic** | **Item #** | **Checklist item** | **Location where item is reported** |
| --- | --- | --- | --- |
| **TITLE** | | |  |
| Title | 1 | Identify the report as a systematic review. | 1 |
| **ABSTRACT** | | |  |
| Abstract | 2 | See the PRISMA 2020 for Abstracts checklist. | 1 |
| **INTRODUCTION** | | |  |
| Rationale | 3 | Describe the rationale for the review in the context of existing knowledge. | 3 |
| Objectives | 4 | Provide an explicit statement of the objective(s) or question(s) the review addresses. | 3 |
| **METHODS** | | |  |
| Eligibility criteria | 5 | Specify the inclusion and exclusion criteria for the review and how studies were grouped for the syntheses. | 4 |
| Information sources | 6 | Specify all databases, registers, websites, organisations, reference lists and other sources searched or consulted to identify studies. Specify the date when each source was last searched or consulted. | 4 |
| Search strategy | 7 | Present the full search strategies for all databases, registers and websites, including any filters and limits used. | 4 |
| Selection process | 8 | Specify the methods used to decide whether a study met the inclusion criteria of the review, including how many reviewers screened each record and each report retrieved, whether they worked independently, and if applicable, details of automation tools used in the process. | 4 |
| Data collection process | 9 | Specify the methods used to collect data from reports, including how many reviewers collected data from each report, whether they worked independently, any processes for obtaining or confirming data from study investigators, and if applicable, details of automation tools used in the process. | 5 |
| Data items | 10a | List and define all outcomes for which data were sought. Specify whether all results that were compatible with each outcome domain in each study were sought (e.g. for all measures, time points, analyses), and if not, the methods used to decide which results to collect. | 5 |
|  | 10b | List and define all other variables for which data were sought (e.g. participant and intervention characteristics, funding sources). Describe any assumptions made about any missing or unclear information. | 5 |
| Study risk of bias assessment | 11 | Specify the methods used to assess risk of bias in the included studies, including details of the tool(s) used, how many reviewers assessed each study and whether they worked independently, and if applicable, details of automation tools used in the process. | 5 |
| Effect measures | 12 | Specify for each outcome the effect measure(s) (e.g. risk ratio, mean difference) used in the synthesis or presentation of results. | 5 |
| Synthesis methods | 13a | Describe the processes used to decide which studies were eligible for each synthesis (e.g. tabulating the study intervention characteristics and comparing against the planned groups for each synthesis (item #5)). | 6 |
|  | 13b | Describe any methods required to prepare the data for presentation or synthesis, such as handling of missing summary statistics, or data conversions. | 6 |
|  | 13c | Describe any methods used to tabulate or visually display results of individual studies and syntheses. | 6 |
|  | 13d | Describe any methods used to synthesize results and provide a rationale for the choice(s). If meta-analysis was performed, describe the model(s), method(s) to identify the presence and extent of statistical heterogeneity, and software package(s) used. | 6 |
|  | 13e | Describe any methods used to explore possible causes of heterogeneity among study results (e.g. subgroup analysis, meta-regression). | 6 |
|  | 13f | Describe any sensitivity analyses conducted to assess robustness of the synthesized results. | 6 |
| Reporting bias assessment | 14 | Describe any methods used to assess risk of bias due to missing results in a synthesis (arising from reporting biases). | 5 |
| Certainty assessment | 15 | Describe any methods used to assess certainty (or confidence) in the body of evidence for an outcome. | 5 |
| **RESULTS** | | |  |
| Study selection | 16a | Describe the results of the search and selection process, from the number of records identified in the search to the number of studies included in the review, ideally using a flow diagram. | 6 |
|  | 16b | Cite studies that might appear to meet the inclusion criteria, but which were excluded, and explain why they were excluded. | 6 |
| Study characteristics | 17 | Cite each included study and present its characteristics. | 6 |
| Risk of bias in studies | 18 | Present assessments of risk of bias for each included study. | 7 |
| Results of individual studies | 19 | For all outcomes, present, for each study: (a) summary statistics for each group (where appropriate) and (b) an effect estimate and its precision (e.g. confidence/credible interval), ideally using structured tables or plots. | 7 |
| Results of syntheses | 20a | For each synthesis, briefly summarise the characteristics and risk of bias among contributing studies. | 7-8 |
|  | 20b | Present results of all statistical syntheses conducted. If meta-analysis was done, present for each the summary estimate and its precision (e.g. confidence/credible interval) and measures of statistical heterogeneity. If comparing groups, describe the direction of the effect. | 7-8 |
|  | 20c | Present results of all investigations of possible causes of heterogeneity among study results. | 7-8 |
|  | 20d | Present results of all sensitivity analyses conducted to assess the robustness of the synthesized results. | 7-8 |
| Reporting biases | 21 | Present assessments of risk of bias due to missing results (arising from reporting biases) for each synthesis assessed. | 8 |
| Certainty of evidence | 22 | Present assessments of certainty (or confidence) in the body of evidence for each outcome assessed. | 8 |
| **DISCUSSION** | | |  |
| Discussion | 23a | Provide a general interpretation of the results in the context of other evidence. | 9 |
|  | 23b | Discuss any limitations of the evidence included in the review. | 10-12 |
|  | 23c | Discuss any limitations of the review processes used. | 10-12 |
|  | 23d | Discuss implications of the results for practice, policy, and future research. | 10-12 |
| **OTHER INFORMATION** | | |  |
| Registration and protocol | 24a | Provide registration information for the review, including register name and registration number, or state that the review was not registered. | 4 |
|  | 24b | Indicate where the review protocol can be accessed, or state that a protocol was not prepared. | 4 |
|  | 24c | Describe and explain any amendments to information provided at registration or in the protocol. | 4 |
| Support | 25 | Describe sources of financial or non-financial support for the review, and the role of the funders or sponsors in the review. | 13 |
| Competing interests | 26 | Declare any competing interests of review authors. | 13 |
| Availability of data, code and other materials | 27 | Report which of the following are publicly available and where they can be found: template data collection forms; data extracted from included studies; data used for all analyses; analytic code; any other materials used in the review. | 13 |

**Table S2. Search strategy**

| Search | Query |
| --- | --- |
| #1 | SARS-COV2 |
| #2 | COVID-19 |
| #3 | 2019-nCoV |
| #4 | 2019-novel coronavirus |
| #5 | FIB-4 |
| #6 | NAFLD fibrosis score |
| #7 | Aspartate aminotransferase to platelet ratio index |
| #8 | Fibrosis |
| #9 | Liver diseases |
| #10 | mechanical ventilation |
| #11 | admission |
| #12 | hospitalization |
| #13 | death |
| #14 | mortality |
| #15 | Severe Covid-19 |
| #16 | Intensive care unit |
| #17 | outcomes |
| #18 | #1 OR #2 OR #3 OR #4 |
| #19 | #5 OR #6 OR #7 OR #8 OR #9 |
| #20 | #10 OR #11 AND #12 OR#13 OR#14 OR#15 OR#16 OR #17 |
|  |  |

**Table S3. Studies excluded with reasons**

| **Studies excluded** | **Reasons** |
| --- | --- |
| Del Zompo F, et al^1^ | This is a meta-analysis of liver injury and clinical outcomes in patients with COVID-19 |
| Anno T, et al^2^ | No target outcomes |
| Campos-Murguia A, et al^3^ | Univariate analysis |
| Chun HS, et al^4^ | No target population: T2DM population |
| Ginès P, et al^5^ | This is a review of liver fibrosis in the general population |
| Goel H, et al^6^ | No target exposure |
| Harris R, et al^7^ | This is a meta-analysis of liver fibrosis in the general population |
| Kim D, et al^8^ | No target exposure: chronic liver disease |
| Lopez-Mendez I, et al^9^ | Univariate analysis |
| Mushtaq K, et al^10^ | No target exposure: Non-alcoholic fatty liver disease |
| Parohan M, et al^11^ | This is a meta-analysis of liver injury and severe COVID-19 infection |
| Pranata R, et al^12^ | This is a meta-analysis |
| Schonmann Y, et al^13^ | No target population: general population |
| Singh A, et al^14^ | This is a meta-analysis of Non-alcoholic fatty liver disease and clinical outcomes in patients with COVID-1 |
| Sy-Janairo ML, et al^15^ | No target exposure: metabolic-associated fatty liver disease |
| Qin C, et al^16^ | Univariate analysis |

coronavirus disease 2019 (COVID-19)

**Supplemental Table S4: Newcastle-Ottawa Scale (NOS) scores for included studies**

| **Author, years** | **selection** | | | | **Comparability** | **Outcome** | | | **Total** |
| --- | --- | --- | --- | --- | --- | --- | --- | --- | --- |
|  | **Exposed cohort** | **None exposed cohort** | **Ascertainment of exposure** | **Outcome of interest** |  | **Assessment of outcome** | **Length of follow-up** | **Adequacy of follow-up** |  |
| Xiang, 2020, China | 1 | 1 | 1 | 1 | 1 | 0 | 1 | 0 | 7 |
| Cristóbal, 2021, Spain | 1 | 1 | 1 | 1 | 1 | 1 | 0 | 0 | 7 |
| Elfeki, 2021, USA | 1 | 1 | 1 | 1 | 1 | 1 | 0 | 0 | 7 |
| Samaniego, 2021, Spain | 1 | 1 | 1 | 1 | 1 | 1 | 0 | 0 | 7 |
| Li, 2021, USA, | 1 | 1 | 1 | 1 | 0 | 0 | 1 | 0 | 7 |
| Calapod, 2020, Romania | 1 | 1 | 1 | 1 | 1 | 1 | 0 | 1 | 8 |
| Forlano, 2020, USA | 1 | 1 | 1 | 1 | 1 | 1 | 0 | 1 | 8 |
| Targher, 2021, China | 1 | 1 | 1 | 1 | 1 | 1 | 1 | 0 | 8 |
| Park, 2020, South Korea | 1 | 1 | 1 | 1 | 1 | 0 | 1 | 0 | 7 |
| Sterlin,2020, USA | 1 | 1 | 1 | 1 | 1 | 1 | 0 | 0 | 7 |
| Rentsch, 2020, UK | 1 | 1 | 1 | 1 | 0 | 1 | 1 | 1 | 8 |
| Yao, 2021, | 1 | 1 | 1 | 1 | 1 | 1 | 1 | 0 | 8 |
| Biliotti, 2020, Italy | 1 | 1 | 1 | 1 | 0 | 1 | 0 | 0 | 6 |
| Fu, 2020, China | 1 | 0 | 1 | 1 | 0 | 0 | 1 | 1 | 7 |
| Sarin, 2020，multinational | 1 | 1 | 1 | 1 | 1 | 1 | 0 | 0 | 7 |
| Goel,2020, USA | 1 | 1 | 1 | 1 | 1 | 0 | 1 | 0 | 7 |

1 Del Zompo, F. *et al.* Prevalence of liver injury and correlation with clinical outcomes in patients with COVID-19: systematic review with meta-analysis. *Eur Rev Med Pharmacol Sci* **24**, 13072-13088, doi:10.26355/eurrev_202012_24215 (2020).

2 Anno, T. *et al.* Marked elevation of plasma procalcitonin levels in patients with diabetic ketoacidosis: A possible useful diagnostic biomarker. *Diabetes Metab* **46**, 504-505, doi:10.1016/j.diabet.2019.05.006 (2020).

3 Campos-Murguia, A. *et al.* Liver fibrosis in patients with metabolic associated fatty liver disease is a risk factor for adverse outcomes in COVID-19. *Dig Liver Dis* **53**, 525-533, doi:10.1016/j.dld.2021.01.019 (2021).

4 Chun, H. S. *et al.* Association between the severity of liver fibrosis and cardiovascular outcomes in patients with type 2 diabetes. *J Gastroenterol Hepatol* **36**, 1703-1713, doi:10.1111/jgh.15387 (2021).

5 Ginès, P. *et al.* Screening for liver fibrosis in the general population: a call for action. *The Lancet Gastroenterology & Hepatology* **1**, 256-260, doi:10.1016/s2468-1253(16)30081-4 (2016).

6 Goel, H. *et al.* The liver in COVID-19: prevalence, patterns, predictors, and impact on outcomes of liver test abnormalities. *Eur J Gastroenterol Hepatol* **Publish Ahead of Print**, doi:10.1097/MEG.0000000000002021 (2020).

7 Harris, R., Harman, D. J., Card, T. R., Aithal, G. P. & Guha, I. N. Prevalence of clinically significant liver disease within the general population, as defined by non-invasive markers of liver fibrosis: a systematic review. *The Lancet Gastroenterology & Hepatology* **2**, 288-297, doi:10.1016/s2468-1253(16)30205-9 (2017).

8 Kim, D. *et al.* Predictors of Outcomes of COVID-19 in Patients With Chronic Liver Disease: US Multi-center Study. *Clin Gastroenterol Hepatol* **19**, 1469-1479 e1419, doi:10.1016/j.cgh.2020.09.027 (2021).

9 Lopez-Mendez, I. *et al.* Association of liver steatosis and fibrosis with clinical outcomes in patients with SARS-CoV-2 infection (COVID-19). *Ann Hepatol* **20**, 100271, doi:10.1016/j.aohep.2020.09.015 (2021).

10 Mushtaq, K. *et al.* NAFLD is a predictor of liver injury in COVID-19 hospitalized patients but not of mortality, disease severity on the presentation or progression - The debate continues. *J Hepatol* **74**, 482-484, doi:10.1016/j.jhep.2020.09.006 (2021).

11 Parohan, M., Yaghoubi, S. & Seraji, A. Liver injury is associated with severe coronavirus disease 2019 (COVID-19) infection: A systematic review and meta-analysis of retrospective studies. *Hepatol Res* **50**, 924-935, doi:10.1111/hepr.13510 (2020).

12 Pranata, R. *et al.* Fibrosis-4 index and mortality in coronavirus disease 2019. *European Journal of Gastroenterology & Hepatology* **Publish Ahead of Print**, doi:10.1097/meg.0000000000002091 (2021).

13 Schonmann, Y., Yeshua, H., Bentov, I. & Zelber-Sagi, S. Liver fibrosis marker is an independent predictor of cardiovascular morbidity and mortality in the general population. *Dig Liver Dis* **53**, 79-85, doi:10.1016/j.dld.2020.10.014 (2021).

14 Singh, A., Hussain, S. & Antony, B. Non-alcoholic fatty liver disease and clinical outcomes in patients with COVID-19: A comprehensive systematic review and meta-analysis. *Diabetes Metab Syndr* **15**, 813-822, doi:10.1016/j.dsx.2021.03.019 (2021).

15 Sy-Janairo, M. L. & IH, Y. C. Association of metabolic-associated fatty liver disease and risk of severe coronavirus disease 2019 illness. *JGH Open*, doi:10.1002/jgh3.12465 (2020).

16 Qin, C. *et al.* High aspartate aminotransferase to alanine aminotransferase ratio on admission as risk factor for poor prognosis in COVID-19 patients. *Sci Rep* **10**, 16496, doi:10.1038/s41598-020-73575-2 (2020).


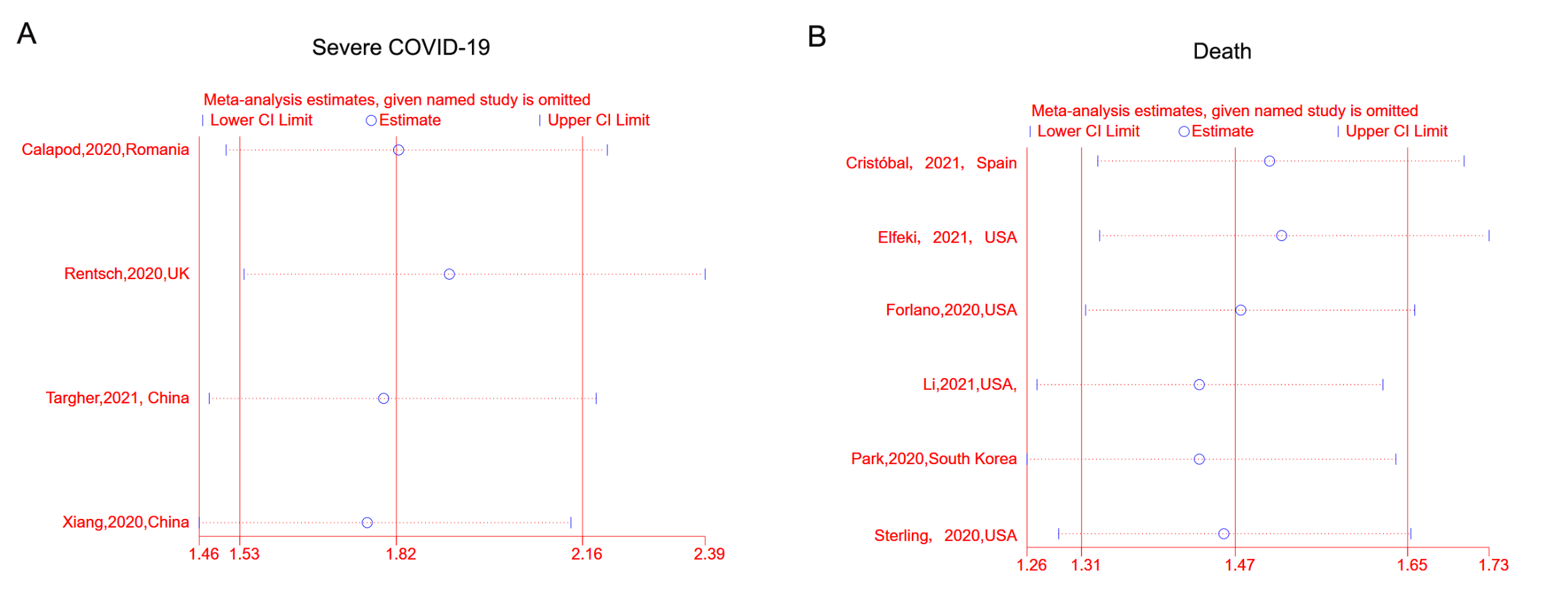


**Figure S1: Sensitive analysis between liver fibrosis scores and clinical outcomes in patients with COVID-19 by omitting one study at each time. A: severe COVID-19; B: Death**
